# Missed transthoracic echocardiography diagnosis of unruptured sinus of Valsalva aneurysm with thrombus: a case report

**DOI:** 10.1093/ehjcr/ytaf201

**Published:** 2025-04-25

**Authors:** Wan-wan Song, Shuang Wang, Gao-feng Pan, Jian-liang Zhou, Bin Wang

**Affiliations:** Department of Cardiovascular Ultrasound, Zhongnan Hospital of Wuhan University, No. 169 of Donghu Road, Wuhan 430071, China; Department of Cardiovascular Ultrasound, Zhongnan Hospital of Wuhan University, No. 169 of Donghu Road, Wuhan 430071, China; Department of Cardiovascular Surgery, Zhongnan Hospital of Wuhan University, No. 169 of Donghu Road, Wuhan 430071, China; Department of Cardiovascular Surgery, Zhongnan Hospital of Wuhan University, No. 169 of Donghu Road, Wuhan 430071, China; Department of Cardiovascular Ultrasound, Zhongnan Hospital of Wuhan University, No. 169 of Donghu Road, Wuhan 430071, China

**Keywords:** Non-coronary sinus of Valsalva aneurysm, Mural thrombus, Transthoracic echocardiography (TTE), Transoesophageal echocardiography (TEE), Coronary computed tomography angiography (CCTA), Thrombectomy, Case report

## Abstract

**Background:**

Unruptured sinus of Valsalva aneurysm (SVA) is a rare congenital or acquired cardiac lesion. It may remain asymptomatic or present with various clinical manifestations due to its ‘mass effect’ on the coronary arteries, valves, atria, and other adjacent structures. Unruptured SVA also carries the risk of secondary complications such as infection, thrombosis, and rupture, posing a serious threat to patient health.

**Case summary:**

A 55-year-old male from the central region of China, who presented with chest tightness following COVID-19 infection, underwent transthoracic echocardiography (TTE) one year before, which revealed an unruptured non-coronary SVA. Recent follow-up TTE showed findings consistent with the previous examination. Coronary computed tomography angiography (CCTA) subsequently demonstrated a non-coronary SVA complicated by a large thrombus and atherosclerotic changes in the aortic sinus wall. Due to the significant risk of thrombus dislodgement and subsequent systemic embolism, the patient underwent surgical thrombectomy and plasty of the non-coronary SVA. Intraoperative transoesophageal echocardiography (TEE) and surgical exploration confirmed the presence of a mural thrombus; while the potential association between COVID-19 infection and aneurysm formation remains to be fully elucidated. At the 6-month postoperative follow-up, the patient demonstrated favourable physical recovery, with no reported thrombo-embolic events (e.g. stroke or systemic embolism), recurrent symptoms, or other adverse clinical outcomes. A repeat TTE during follow-up revealed a well-preserved aortic root morphology, with normal dimensions and no signs of residual aneurysm or valvular dysfunction.

**Discussion:**

We report a unique instance of a massive thrombosed non-coronary SVA. Considering the limitations of TTE and its low sensitivity to SVA secondary lesions, it is recommended to further perform TEE or CCTA to comprehensively evaluate the morphology and structure of SVA.

**Learning points**

- Transthoracic echocardiography (TTE) is the first-line imaging modality for evaluating and monitoring sinus of Valsalva aneurysms (SVAs). However, suboptimal acoustic windows, vessel wall calcification, or other inherent limitations of TTE may reduce its sensitivity in detecting lesions.
- Management of SVA should incorporate multimodality cardiovascular imaging modalities [e.g. TTE, transoesophageal echocardiography (TEE), coronary computed tomography angiography, or others] to thoroughly evaluate SVA morphology and its ‘mass effect’ on adjacent anatomical structures.
- Intraoperative TEE facilitates surgical decision-making and enables immediate assessment of postoperative.

## Introduction

Sinus of Valsalva aneurysm (SVA) is an infrequent congenital or acquired anomaly, with an incidence of around 0.09% in the general population. It typically refers to the dilation of one of the three aortic sinuses.^1,2^ Sinus of Valsalva aneurysm is caused by fibrosis of the annulus and weakening of the elastic layer in the aortic media. It is frequently associated with congenital heart diseases, such as ventricular septal defect and bicuspid aortic valve, and connective tissue pathologies like Marfan syndrome, or other aetiological factors.^2^ Sinus of Valsalva aneurysm can also result from iatrogenic damage, atherosclerosis, or infections such as bacterial endocarditis, syphilis, tuberculosis, or COVID-19.^3,4^ The most characteristic outcome of SVA is rupture, which may lead to symptoms ranging from mild to severe dyspnoea or chest pain. Patients who do not undergo timely surgery face a high risk of death. Conversely, unruptured SVA is usually asymptomatic or causes various clinical manifestations due to the ‘mass effect’ on adjacent structures. It also carries the risks of secondary infection, thrombosis, rupture, and other complications, seriously threatening patients’ health.^5^ In this instance, we present a middle-aged man with chest tightness who had been experiencing an unruptured SVA for over a year.

## Summary figure

**Table:** 

1 year prior to presentation
Presented with chest tightness; COVID-19 infection suspected
Electrocardiogram (ECG) and chest radiograph were normal.
Transthoracic echocardiography (TTE) revealed a non-coronary sinus of Valsalva aneurysm (SVA) measuring 5.0 cm.
Medical history: hypertension for 10 years; smoking history of 30 years (20 cigarettes/day)
Follow-up visit
Asymptomatic; undergoing surveillance for non-coronary SVA
ECG: first-degree atrioventricular block
TTE: SVA diameter stable at 5.1 cm (similar to prior measurement)
Coronary computed tomography angiography (CCTA): non-coronary SVA with thrombus formation
Brain CT: left paraventricular infarction
Admission and surgical planning (Day 0)
Planned for thrombectomy and repair of the non-coronary SVA
Postoperative Day 7
TTE: aortic sinus diameter reduced to 3.8 cm; no aortic regurgitation detected
Patient recovered adequately and was discharged.
6 months postoperatively
Full physical recovery achieved
Successfully quit smoking
TTE findings were nearly identical to those on postoperative Day 7.

L, left; R, right; N, non; AS, aortic sinus; CCTA, coronary computed tomography angiography; ECG, electrocardiogram; TEE, transoesophageal echocardiography; TTE, transthoracic echocardiography; SVA, sinus of Valsalva aneurysm.

## Case presentation

A 55-year-old man presented to our hospital one year ago with chest tightness, initially suspected to be related to COVID-19 infection. Despite a 10-year history of hypertension, the patient had been in generally good health. He was prescribed amlodipine 5 mg daily—a calcium channel blocker for antihypertensive therapy—but did not regularly monitor his blood pressure. With a 30-year smoking history (20 cigarettes per day), his physical examination, electrocardiogram (ECG), and chest radiograph findings were unremarkable. Transthoracic echocardiography (TTE) demonstrated a non-coronary SVA measuring 5.0 cm without aortic regurgitation. Given that the SVA did not meet surgical indications, the physician advised smoking cessation, blood pressure control, and scheduled follow-up appointments.

**Figure 1:**
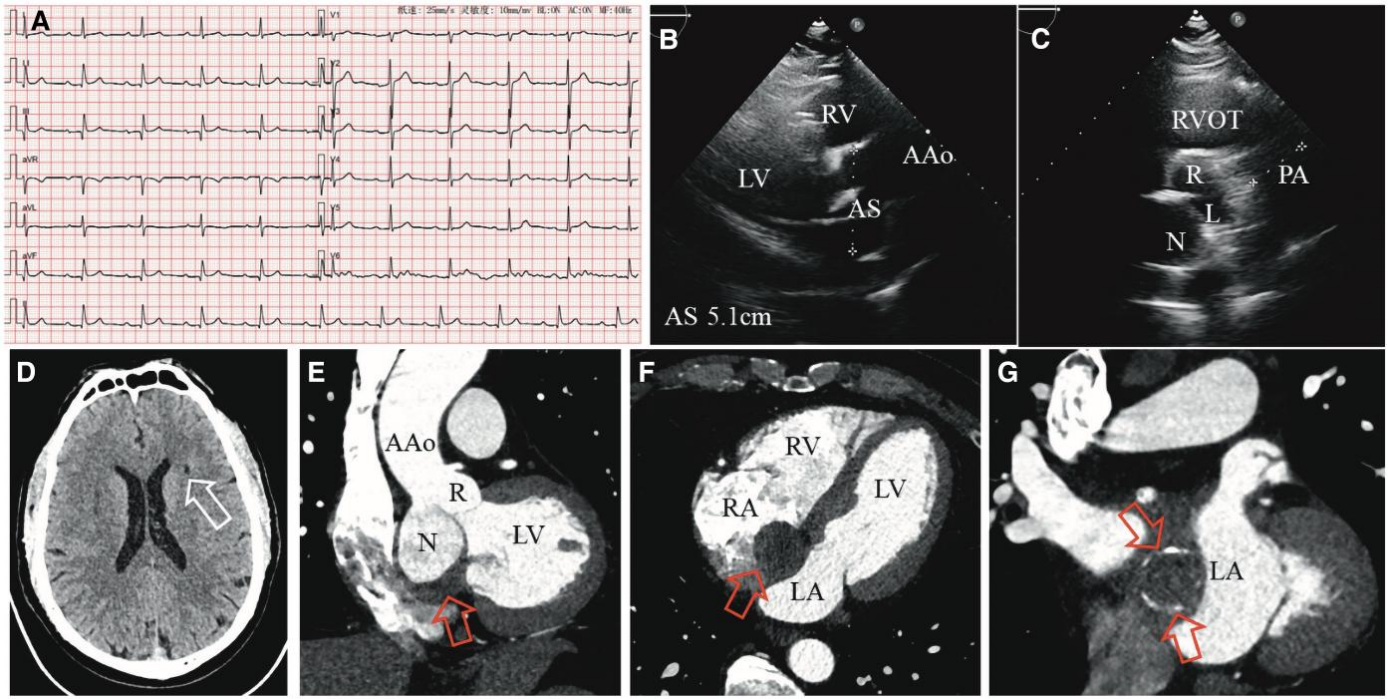
(*a*) The ECG showed first-degree atrioventricular block. (*b* and *c*) Transthoracic echocardiography showed a non-coronary SVA. (*d*) Brain CT indicated infarction of the left paraventricular region. (*e–f*) Mural thrombus of a non-coronary SVA was observed from multiple perspectives in CCTA. (*g*) Circular calcification of the non-coronary sinus wall.

Recently, the patient returned to our hospital for a follow-up. Over the past year, he occasionally experienced chest tightness, which was relieved after rest. His blood pressure was well controlled, but he continued to smoke 20 cigarettes per day. Physical examination was normal, and the ECG showed first-degree atrioventricular block (*Figure 1a*). We hypothesized that this might be caused by the ‘mass effect’ of the SVA on the atrial septum or atrioventricular node.^6^ Transthoracic echocardiography showed a similar result as before, with a non-coronary SVA (5.1 cm) and no aortic regurgitation (*Figure 1b* and *c*). Considering the patient’s intermittent chest tightness and continuous smoking history, we conducted relevant blood tests and coronary artery imaging evaluations for him. Blood tests revealed elevated triglycerides (3.21 mmol/L, normal value: <1.70 mmol/L), uric acid (561 µmol/L, normal range: 208–428 µmol/L), and D-dimer (642 ng/mL, normal value: 0–500 ng/mL), while other parameters were normal. Coronary computed tomography angiography (CCTA) showed a non-coronary SVA accompanied by a large mural thrombus and significant calcification of the vascular wall (*Figure 1e–g*). Given the patient’s medical history of chronic hypertension, a non-coronary SVA complicated by a large mural thrombus, which factors collectively posed a considerable risk for cerebrovascular occlusion. A brain CT was ordered subsequently, which showed a left paraventricular infarction (*Figure 1d*).

**Figure 2:**
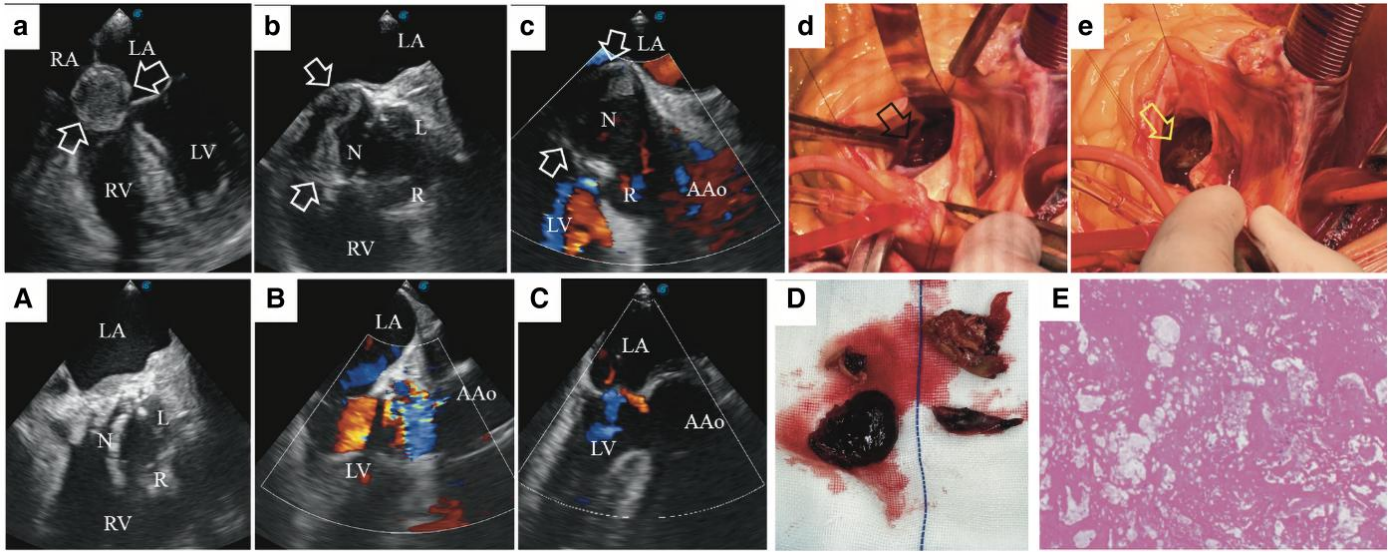
(*a–c*) Mural thrombus of a non-coronary SVA (white arrows) was observed from multiple perspectives in TEE, along with slight aortic regurgitation. (*d*) Thrombus *in situ* (black arrow). (*e*) Atherosclerotic aortic sinus wall after thrombus removal (yellow arrow). (*A–C*) Postoperative TEE showed non-coronary sinus plasty. (*D* and *E*) Gross specimen of chronic and fresh thrombus, confirmed by histological examination.

Systemic embolism risk is significantly elevated in SVA complicated by thrombosis, and thrombus formation is recognized as an established surgical indication.^1^ According to current guidelines, this indication is classified as Class I (strong recommendation) with Level C-LD evidence.^7^ Given the indications for surgery, the physician advised the patient to undergo hospitalization for definitive surgical management, to which the patient consented. During the operation, before clamping the ascending aorta, intraoperative transoesophageal echocardiography (TEE) showed a non-coronary SVA complicated with mural thrombus, with the aortic valve functioning well and the other two sinuses having normal morphology (*Figure 2a–c* and *A–C*, Supplementary material online, *Videos S1*–*S5*). After thrombus removal, the surgeons elected to repair the non-coronary SVA by continuously suturing a 3 × 4 cm patch-trimmed from a prosthetic vessel-starting at the base of the non-coronary sinus. Subsequently, the prosthetic vessel was wrapped with autologous tissue and anastomosed to the ascending aorta. Following the establishment of stable cardiac circulation, intraoperative TEE was performed to systematically assess the adequacy of the surgical repair. Results indicated that the post-repair morphology of the non-coronary sinus was symmetric relative to the remaining sinuses, with preserved aortic valve competency. The thrombus was a mixed type, with the ‘head’ attached to the vascular wall, composed of white thrombus, and the ‘tail’ composed of red thrombus, which was confirmed by histological examination. With the thrombus completely removed, the vascular wall showed severe atherosclerosis (*Figure 2d* and *e*, *D* and *E*). Based on the TEE assessment of the structure and function of the aortic root, the surgeon decided to reconstruct the non-coronary sinus rather than replace the aortic root. The operation proceeded smoothly. The patient recovered well and was discharged from the hospital one week after surgery. Six months postoperatively, the patient achieved full physical recovery, and TTE findings were nearly identical to those on postoperative Day 7. Additionally, the patient successfully quit smoking.

## Discussion

We report a rare case of an unruptured non-coronary SVA complicated with mural thrombus. Three aspects of this case are particularly notable.

Firstly, while TTE is the first-line imaging modality for assessing and monitoring SVA, it has inherent limitations that may compromise its sensitivity in lesion detection.^1^ Upon case review, one contributing factor to the undetected thrombus on TTE was vascular wall calcification, which obscured the thrombus within the hyperechoic vessel wall. Coronary computed tomography angiography and TEE can compensate for the deficiencies of TTE. However, TEE is still an invasive operation, and during intubation or when sedation is inadequate, a rise in systemic pressure from retching and gagging may pose a potential theoretical risk for rupture in patients with SVA, and even carry the risk of detachment of mural thrombi.^1^ Therefore, we performed intraoperative TEE to evaluate the aortic valve and the adjacent structures of the SVA and exclude other intracardiac structural abnormalities, such as small atrial or ventricular septal defects, which are also easily missed by TTE or CCTA. Intraoperative TEE can effectively guide surgeons’ decision-making and evaluate the immediate postoperative effects, including the effect of SVA plasty and the function of the aortic valve. The principal advantage of CCTA lies in its superior spatial resolution, enabling precise delineation of aortic root pathology and accurate assessment of coronary artery disease.^1,3^ This imaging modality serves as a valuable complementary diagnostic tool to TEE, particularly in cases where TEE findings are inconclusive or require further anatomical clarification.

Secondly, an in-depth analysis of the potential aetiologies of SVA thrombosis was performed. By integrating the patient’s history of long-term smoking, hypertension, and previous COVID-19 infection, together with laboratory findings revealing elevated triglycerides and uric acid-both independent risk factors for atherosclerosis-and increased D-dimer levels indicative of thrombotic and hyperfibrinolytic states, and considering the atherosclerotic alterations in the vessel wall confirmed by imaging and surgical observations, we postulate that SVA thrombosis stemmed from coagulopathy and impaired fibrinolysis triggered by endotheliitis and the associated cytokine storm within the non-coronary sinus wall. While a potential link to COVID-19 infection exists, the exact nature of this association remains undetermined.^4,8^ To date, only a limited number of comparable cases have been reported. The left paraventricular infarction detected on brain CT is likely attributable to chronic hypertension and smoking.

Finally, current therapeutic modalities for SVA encompass transcatheter and surgical repair strategies.^5^ The presence of mural thrombus constitutes a contraindication for percutaneous transcatheter approaches, in accordance with the 2022 ACC/AHA Guideline recommendations for open surgical therapy.^7^ Surgical challenges include the decision-making regarding aortic root and valve reconstruction vs. replacement, as well as potential postoperative sequelae. In this case, intraoperative TEE can effectively mitigate these concerns.

## Conclusions

The outcome of this case was favourable. Transthoracic echocardiography is the first-line imaging modality for the assessment and follow-up of SVA, but it has limitations, especially in cases with poor acoustic windows, calcified vessel walls, or other factors. Coronary computed tomography angiography and TEE can complement TTE. Coronary computed tomography angiography can comprehensively evaluate the morphology of SVA and its ‘mass effect’ on adjacent structures. Transoesophageal echocardiography, particularly intraoperative TEE, plays a crucial role in surgical decision-making and the evaluation of immediate postoperative effects.

## Supplementary Material

ytaf201_Supplementary_Data

## Data Availability

All data are incorporated into this article and its online Supplementary material.

## Lead author biography

Dr Wan-wan Song is a graduate of Department of Ultrasound Imaging, Renmin Hospital of Wuhan University, where she earned her MD degree and finished a residency programme in 2020. Following this, she is an echocardiologist in Department of Cardiovascular Ultrasound, Zhong Nan Hospital of Wuhan University, China.

## Supplementary material

**Consent:** The authors confirm that both written and verbal informed consent for the submission and publication of this case report including any associated text and images has been obtained from the patient in accordance with COPE guidelines.

**Funding:** Hubei Provincial Natural Science Foundation - Joint Fund (2025AFD873).
